# Impact of age at first visit on glycolipid metabolism, bone metabolism, and fertility potential in patients with Klinefelter syndrome

**DOI:** 10.1111/andr.70033

**Published:** 2025-04-07

**Authors:** Giordana Ferraioli, Andrea Graziani, Noemi Sagone, Andrea Di Nisio, Massimo Iafrate, Fabrizio Dal Moro, Alberto Ferlin, Andrea Garolla

**Affiliations:** ^1^ Unit of Andrology and Reproductive Medicine, Department of Systems Medicine University Hospital of Padova Padova Italy; ^2^ Department of Medicine University of Padova Padova Italy; ^3^ Unit of Urology, Department of Surgery, Oncology and Gastroenterology University Hospital of Padova Padova Italy

**Keywords:** age, bone, fertility, Klinefelter syndrome, metabolism, testicular biopsy

## Abstract

**Background:**

Classic Klinefelter syndrome (KS) is characterized by one extra X chromosome (47, XXY), leading to hypergonadotropic hypogonadism and higher risk of alterations in glycolipid homeostasis, cardiovascular diseases, and low bone mineral density. Most frequently, KS is diagnosed in adulthood because of infertility.

**Objectives:**

To investigate the potential association between the age at first visit and the presence of comorbidities in patients with KS.

**Materials and methods:**

In this cross‐sectional retrospective study, we analyzed the data from 445 patients affected by non‐mosaic 47, XXY KS and aged less than 50 years. Anthropometric measurements, biochemical and hormonal tests, semen analysis, scrotal echo‐color Doppler, and dual energy X‐ray absorptiometry (DXA) were performed on the patients. A subset of patients underwent testicular sperm extraction (TESE).

**Results:**

Age at first visit significantly correlated positively with waist circumference (WC), body mass index (BMI), blood glucose, glycated hemoglobin, total cholesterol, low‐density lipoprotein (LDL) cholesterol, and triglycerides, and negatively with total testosterone (TT), calculated free testosterone (cFT), calcium, phosphorus, vitamin D, and lumbar *Z*‐score. Age at the first visit >26 years was associated with higher frequency of increased WC, BMI, and hypercholesterolemia. Among the 199 patients who underwent testicular biopsy, the mean retrieval rate was 36.2% and was higher in the younger group (<26 years).

**Discussion and conclusion:**

Early diagnosis and management of KS is important for preventing or reducing comorbidities later in life. In particular, glycemic, lipid, and phospho‐calcium metabolism worsen with advancing age at first visit. Furthermore, early management of the patient seems to be associated with a higher probability of recovering spermatozoa through TESE.

## 1 INTRODUCTION

First described in 1942, Klinefelter syndrome (KS) is caused by a numerical abnormality of sex chromosomes and is characterized by the presence of two or more X chromosomes in male subjects.^1^ KS is the most frequent chromosomal aberration, with an incidence of about 1:500 males, although it is likely underestimated due to its blurry phenotype leading to missed diagnosis.^2^, ^3^ The delay or lack of diagnosis presents significant problems that should not be underestimated; the diagnostic delay could strongly impact the outcome of testosterone replacement therapy (TRT) and assisted reproductive techniques (ART).^4^

The classic genotype (non‐mosaic), 47, XXY, results from the missed disjunction of sex chromosomes during gametogenesis.^1^, ^2^ While the classic genotype is most common, some patients exhibit mosaicism (46, XY/47, XXY), higher grade aneuploidies, or structurally abnormal X chromosomes.^2^

The clinical presentation depends on both the presence of extra X chromosome and hypogonadism, which is a key element of the syndrome.^2^ Although hormone levels might be normal during infancy, a decline in testicular function generally occurs at mid‐puberty, leading to reduced testosterone production and primary hypogonadism. Consequently, there is an increase in serum concentration of luteinizing hormone (LH) and follicular stimulating hormone (FSH), which together with reduced testicular volumes are frequently the sole hallmark of the syndrome.^4^

Spermatogenesis is altered or completely absent, resulting in azoospermia in approximately 90% of patients with the classic genotype 47, XXY.^1^, ^5^ It has been demonstrated previously that a reduction in the number of spermatogonia occurs at the testicular level from prepubertal onward, due to progressive apoptosis, leading to severe oligozoospermia or azoospermia.^6^ However, even azoospermic patients with KS might have foci with residual spermatogenesis.^7^ For this reason, infertility is no longer considered an untreatable condition in KS. In fact, testicular sperm extraction (TESE), in combination with assisted reproduction techniques (ART), has enabled infertility treatment in KS patients, similarly to non‐KS infertile men with non‐obstructive azoospermia.^1^, ^8^, ^9^

KS is also associated with increased risk of alterations in glucose homeostasis,^10^, ^11^ bone mineral density (BMD),^12^ cardiovascular comorbidities,^13^ venous thromboembolism,^14^ malignant neoplasms,^15^, ^16^ and autoimmune diseases.^17^ Glucose homeostasis alterations, such as glucose intolerance, insulin resistance, and metabolic syndrome, have been related to alterations in body composition. KS patients, in fact, have a higher percentage of truncal fat than control patients for any given body mass index (BMI) value, even in the presence of BMI in the normal range.^18^ Reduced BMD values have been attributed to testosterone deficiency, hypovitaminosis D, lower concentration of insulin‐like factor 3 (INSL‐3), and alterations in body composition, which are frequently observed in these subjects.^19^

Previous studies suggested that early diagnosis and taking care of KS subjects may facilitate the prompt identification of metabolic aberrations and bone health issues, such as dyslipidemia and osteoporosis and prevent their complications.^20^, ^21^ Additionally, timely evaluation and management of fertility potential are vital for addressing reproductive challenges associated with the syndrome. Recognizing the influence of age‐related factors on diagnosis and initial assessment is crucial for devising effective interventions and optimizing long‐term health outcomes in KS subjects.

The present study aims to investigate the impact of age at the time of the first visit on glycolipid and bone metabolism and fertility potential.

## 2 MATERIALS AND METHODS

This cross‐sectional retrospective study included 445 patients affected by KS with the classic 47, XXY genotype and less than 50 years of age, attending the Unit of Andrology and Reproductive Medicine at the University‐Hospital of Padua. The patients were referred to our Unit because of pubertal delay, poor androgenization, gynecomastia, infertility, hypogonadism, osteopenia/osteoporosis, or previous KS diagnosis. To evaluate impact of age, patients were divided into three groups according to tertiles based on the age at the first visit: group A <26 years (*n* = 145, 32.6%), group B ≥26 < 35 years (*n* = 143, 32.1%), and group C ≥35 years (*n* = 157, 35.3%). At the time of the first visit, all patients underwent a medical history and physical examination, including measurements of weight, height, and waist circumference (WC). WC >94 cm was considered pathological. BMI was calculated as body weight in kilograms divided by height in square meters. BMI ≥ 25 kg/m was considered pathological (indicative of overweight or obesity when >30).

Most of the patients underwent biochemical, hormonal, and metabolic assays. Dual energy X‐ray absorptiometry (DXA) was performed on 355 patients to assess BMD values in the lumbar spine and femur.

In azoospermic KS subjects, expressing a desire for paternity, TESE for sperm retrieval with possible cryopreservation was recommended. Among the 445 patients, 199 underwent this procedure.

### 2.1 Hormonal and biochemical assays

Serum concentrations of LH (IU/L), FSH (IU/L), total testosterone (TT; nmol/L), sex hormone binding globulin (SHBG; nmol/L), albumin (g/L), estradiol (pg/mL), gamma glutamyl transferase (GGT; U/L), glucose (mg/dL), glycated hemoglobin (Hb1Ac; %), insulin (mU/L), total cholesterol (mg/dL), low‐density lipoprotein (LDL) cholesterol (mg/dL), high‐density lipoprotein (HDL) cholesterol (mg/dL), triglycerides (mg/dL), homocysteine (umol/L), parathormone (PTH; ng/L), calcium (mmol/L), phosphorous (mmol/L), prostatic specific antigen (PSA; ug/L), and 25‐hydroxy vitamin D (nmol/L) were measured in the same laboratory.

The diagnosis of hypogonadism was based on the presence of clinical symptoms (reduced libido, decreased spontaneous erections, or fatigue), along with impaired spermatogenesis, consistent with low testosterone levels, confirmed by serum testosterone measurements on two separate occasions. TT levels were measured in morning fasting blood samples using a well‐validated assay. A cut‐off value of <8 nmol/L was used to confirm testosterone deficiency. For patients with TT levels 8–12 nmol/L, calculated free testosterone (cFT) from TT, albumin, and SHBG concentration was determined using the Vermeulen equation. Low free testosterone <225 pmol/L was considered as an additional diagnostic criterion. Serum glucose levels of 100–125 mg/dL were considered indicative of impaired fasting glucose, while a diagnosis of diabetes was established if glucose was ≥126 mg/dL and/or Hb1Ac >6.5%. The homeostatic model assessment (HOMA) index was calculated based on glycemia and insulinemia and considered pathological if >2.4. LDL >116 mg/dL was considered hypercholesterolemia; triglycerides >150 mg/dL were considered hypertriglyceridemia; homocysteine >15 umol/L was considered hyperhomocysteinemia. 25‐Hydroxy vitamin D levels <75 nmol/L were considered hypovitaminosis D.

### 2.2 Semen analyses

Semen evaluations were conducted according to the guidelines specified in the fifth and sixth editions of the World Health Organization (WHO) manual for the analysis and processing of human semen.^22^, ^23^

The participants provided semen samples by masturbation into sterile containers after 2–7 days of sexual abstinence. Semen specimens were collected at the Laboratory Medicine Unit of the University Hospital of Padova and maintained at 37°C for at least 30 min before analysis.

The analyses included the measurement of semen volume (mL), pH, sperm concentration (10^6^ cells/mL), total sperm count (10^6^ cells/ejaculate). Sperm concentration (10⁶ cells/mL) was assessed using an improved Neubauer hemocytometer after dilution in a formalin‐based buffer. To ensure accuracy and minimize random errors, at least two 50‐µL aliquots were examined for each sample.

In cases where no spermatozoa were observed in the initial microscopic examination of a 10‐µL aliquot of fresh semen, the sample was centrifuged at 3000 × *g* for 15 min.^24^ The pellet was then carefully resuspended and examined using phase‐contrast microscopy at 200× magnification to detect the presence of rare spermatozoa. This evaluation was performed on at least two separate aliquots to reduce the risk of erroneous results and ensure the accurate diagnosis of the complete absence of spermatozoa in the ejaculate defined as azoospermia.

All analyses adhered to standard procedures at the Laboratory Medicine Unit of the University Hospital of Padova and were performed by the same experienced operator.

### 2.3 Testicular color Doppler ultrasonography

Testicular color Doppler ultrasonography (CDUS) scrotal evaluation was performed using an AplioTM XV echo‐color Doppler device (Toshiba) with a high‐resolution multifrequency linear probe (6–13 MHz). The testicular volume was estimated using the ellipsoid formula (length × width × height × 0.52). The sum of the right and the left testicular volume was defined as bitesticular volume (mL).

### 2.4 Dual energy X‐ray absorptiometry

BMD was assessed using DXA using fan‐beam technology (Hologic QDR 4500W, Inc.) to analyze three areas: lumbar spine, femur, and neck of femur. Since all patients were younger than 50 years, *Z*‐score was used in accordance with the WHO recommendation.^25^ A *Z*‐score lower than −2.0 was considered pathological.

### 2.5 Testicular sperm extraction

TESE was performed under sedation with spermatic cord block with 0.5% bupivacaine solution. After transverse incision of the tunica albuginea on the anterior surface of the testis, two small specimens, each approximately 5 mm in diameter, were excised from each testis using sharp scissors. Testis specimens were placed in a Petri dish with 2 mL of Biggers, Whitten, and Whittingham (BWW) medium (Irvine Scientific), mechanically sectioned by means of sterile slides and then further gently minced by sterile needles. After testicular shredding, the testicular suspension was vortexed for 5 min and then transferred into a 15‐mL conical tube containing 2 mL of fresh medium and centrifuged at 1200 × *g* for 10 min. The supernatant was then discarded, and the pellet was suspended with 1 mL of BWW medium and vortexed for 2 min. Under an inverted microscope at ×400 magnification, the testicular suspension was checked for the presence of mature spermatozoa. If spermatozoa were found, the sample was frozen for future IntraCytoplasmic Sperm Injection (ICSI) use.

All parameters were evaluated both considering all KS patients as whole and distinguishing them based on whether (TRT) or not (no‐TRT) they were undergoing TRT.

### 2.6 Statistical analysis

Statistical analysis of the data was conducted with “Statistical Package for the Social Sciences software IBM SPSS Statistics, Version 29.0.” Continuous variables were expressed as medians and 5th–95th percentiles while categorical variables were expressed as absolute and relative frequencies. The Kolmogorov–Smirnov test was used to check for normality of distribution. Since the majority of variables did not show normal distribution, correlation analysis was performed using the non‐parametric Spearman's test. Pearson's chi‐square test, or Fisher's exact test when the expected frequency was five or less, was used to examine the differences between groups for categorical variables. Pairwise comparisons for continuous variables between groups were performed using the Wilcoxon rank test with Bonferroni–Holm correction for multiple comparisons. Two‐sided *p* values <0.05 were considered statistically significant.

## 3 RESULTS

The clinical characteristics of the 445 patients with non‐mosaic KS are listed in Table 1. The median age at the first visit was 31 (16–45, 95th confidence intervals [CIs]) years. We collected information about smoking habits from 420 patients: 176 patients (39.6%) reported being smokers at the time of the anamnesis while 244 patients (58.1%) reported either never having smoked or having quit since at least 6 months. Diabetes was found in only eight patients (1.8%). Seventy‐six patients (17.1%) were under TRT or had suspended it for less than 6 months, while 77 patients (17.3%) had suspended it since at least 6 months; finally, 292 patients (65.6%) declared that they had never undertaken replacement therapy. Hypogonadism was identified in 59.0% of the population, involving 263 individuals, specifically 224 patients not on therapy (first assessed at our hospital, with KS diagnosed elsewhere) and 39 subjects on substitution therapy. Additionally, some patients were receiving replacement therapy despite not meeting the biochemical criteria for testosterone deficiency. Of these, the majority were treated for borderline testosterone levels associated with clinical symptoms, while others had already initiated therapy prior to their first visit to our hospital, having been previously followed at other institutions (Table S1).

**TABLE 1:** Anthropometric, hematochemical, scrotal ultrasound, and DEXA (Dual Energy X‐ray Absorptiometry) parameters of patients with non‐mosaic Klinefelter syndrome (KS), expressed as median and 5th–95th percentiles.

	*N*	Median	Percentiles	
			5th	95th
Age at first visit	445	31.0	16.0	45.0
Waist circumference (cm)	345	95.0	72.0	109.7
BMI (kg/m^2^)	399	24.80	17.62	31.80
LH (U/L)	417	19.00	4.03	35.13
FSH (U/L)	412	31.85	4.86	56.04
TT (nmol/L)	445	10.05	2.22	19.50
Hypogonadism (%)	263 (59%)			
cFT (nmol/L)	283	0.192	0.034	0.398
SHBG (nmol/L)	335	32.0	14.0	63.0
Albumin (g/L)	337	48.0	43.0	52.0
Estradiol (pg/mL)	399	95.0	25.0	170.0
Glucose (mg/dL)	395	81.0	65.0	101.0
Hb1Ac (%)	380	5.4	4.8	5.9
Diabetes	8 (1.8%)			
Insulin (mU/L)	367	10.8	2.0	20.0
HOMA	366	1.79	0.38	6.85
Total cholesterol (mg/dL)	398	180.0	125.0	252.1
LDL (mg/dL)	397	113.0	68.0	178.1
HDL (mg/dL)	395	48.0	31.8	70.2
Hypercholesterolemia (%)	179 (40.2%)			
Triglyceride (mg/dL)	389	106.0	51.0	198.5
Homocysteine (umol/L)	367	12.6	8.1	33.8
PTH (ng/L)	384	38.0	13.1	65.5
Calcium (mmol/L)	394	2.44	2.30	2.59
Phosphorous (mmol/L)	374	0.95	0.66	1.36
PSA (ug/L)	387	0.65	0.14	1.30
Vitamin D (nmol/L)	374	56.0	19.0	105.8
Bitesticular volume (mL)	388	4.00	1.80	6.70
Prostatic volume (mL)	354	17.0	9.0	27.3
Lumbar BMD	351	1.000	0.782	1.247
Femoral BMD	342	0.978	0.787	1.212
Testosterone replacement therapy (%)	76 (17.1%)			
Smoking habits (%)	176 (39.6%)			

Abbreviations: BMD, bone mineral density; BMI, body mass index; cFT, calculated free testosterone; FSH, follicular stimulating hormone; Hb1Ac, glycosylated hemoglobin; HDL, high‐density lipoprotein; HOMA, homeostatic model assessment; LDL, low‐density lipoprotein; LH, luteinizing hormone; PSA, prostatic specific antigen; PTH, parathormone; SHBG, sex hormone binding globulin; TT, total testosterone.

A comparative analysis between hypogonadal KS patients receiving substitution therapy and those untreated revealed significant differences. Patients in the hypogonadal TRT group showed lower LH (15.70 (0.10–32.84, 95th CIs) vs. 19.00 (8.50–36.32, 95th CIs); *p* = 0.001) and FSH levels (21.40 (0.16–45.66, 95th CIs) vs. 30.25 (11.99–54.31, 95th CIs); *p* < 0.001), higher lumbar (1.067 (0.774–1.350, 95th CIs) vs. 0.989 (0.769–1.279, 95th CIs); *p* = 0.013) and femoral BMD (1.108 (0.837–1.324, 95th CIs) vs. 0.958 (0.777–1.229, 95th CIs); *p* < 0.001), and lower HDL cholesterol levels (41.0 (25.0–76.5, 95th CIs) vs. 48.0 (32.0–71.6, 95th CIs); *p* = 0.014) compared with the untreated group. These findings highlight the potential positive effects of testosterone therapy in this population.

Table 2 shows that age at first visit was significantly related to increases in WC, BMI, blood glucose, glycated hemoglobin, total cholesterol, LDL cholesterol, and triglycerides (all *p* < 0.05). Additionally, it was correlated with significant decreases in both total and cFT, blood calcium, phosphorus, vitamin D, and lumbar *Z*‐score.

**TABLE 2:** Correlations between age at first visit and anthropometric, glyco‐lipidic and phospho‐calcic variables.

	*p* value	*r* value
Waist circumference (cm)	<0.001	0.452
BMI (kg/m^2^)	<0.001	0.383
TT (nmol/L)	<0.001	−0.227
cFT (nmol/L)	<0.001	−0.244
Albumin (g/L)	<0.001	−0.200
Glucose (mg/dL)	<0.001	0.189
Hb1Ac (%)	<0.001	0.205
Total cholesterol (mg/dL)	<0.001	0.520
LDL (mg/dL)	<0.001	0.518
Triglyceride (mg/dL)	<0.001	0.215
PTH (ng/L)	0.001	0.167
Calcium (mmol/L)	<0.001	−0.210
Vitamin D (nmol/L)	<0.001	−0.243
Lumbar *Z*‐score	0.014	−0.129

Abbreviations: BMI, body mass index; cFT, calculated free testosterone; Hb1Ac, glycosylated hemoglobin; LDL, low‐density lipoprotein; PTH, parathormone; *r*, Spearman correlation coefficient; TT, total testosterone.

The study population parameters were reassessed dividing KS patients based on whether they were undergoing TRT (Table S2). The TRT group included patients who were currently on TRT or who had suspended it within the last 6 months, while the group no TRT included those who had never undergone therapy or had discontinued it for at least 6 months. The multivariate analysis, after adjusting for confounding factors, showed that the variables LH, FSH, TT, free testosterone (cfT), and SHBG were significantly different between TRT and no‐TRT patients (*p* < 0.001).

In the correlation analysis between age at first visit and parameters, the same results observed in the whole population were confirmed in both subgroups (TRT, no TRT) except for HDL, phosphorous, lumbar, and femoral BMD that were significantly different in TRT patients.

**FIGURE 1:**
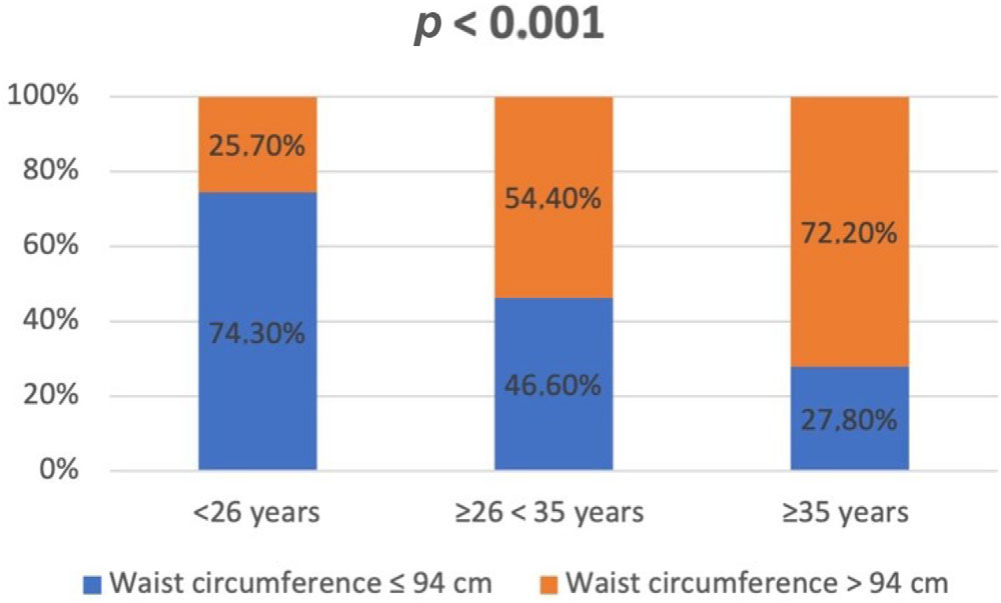
Frequency (%) of increased waist circumference (>94 cm) in the three groups of patients, stratified according to the age at first visit.

**FIGURE 2:**
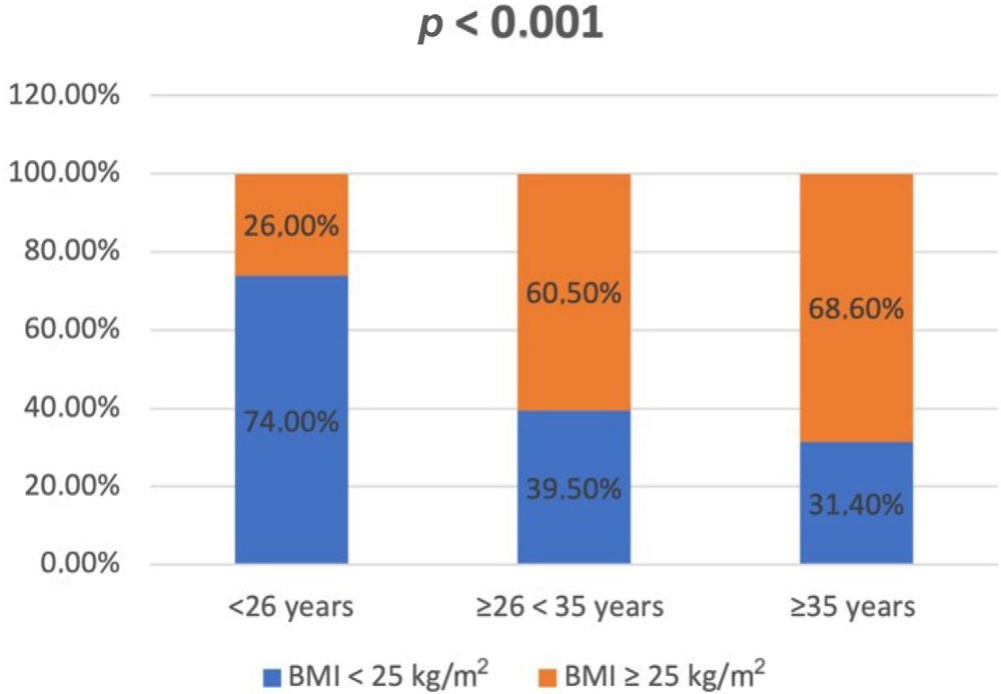
Frequency (%) of overweight (body mass index [BMI] ≥ 25 kg/m^2^) and obesity (BMI ≥ 30 kg/m^2^) in the three groups of patients, stratified according to the age at first visit.

**FIGURE 3:**
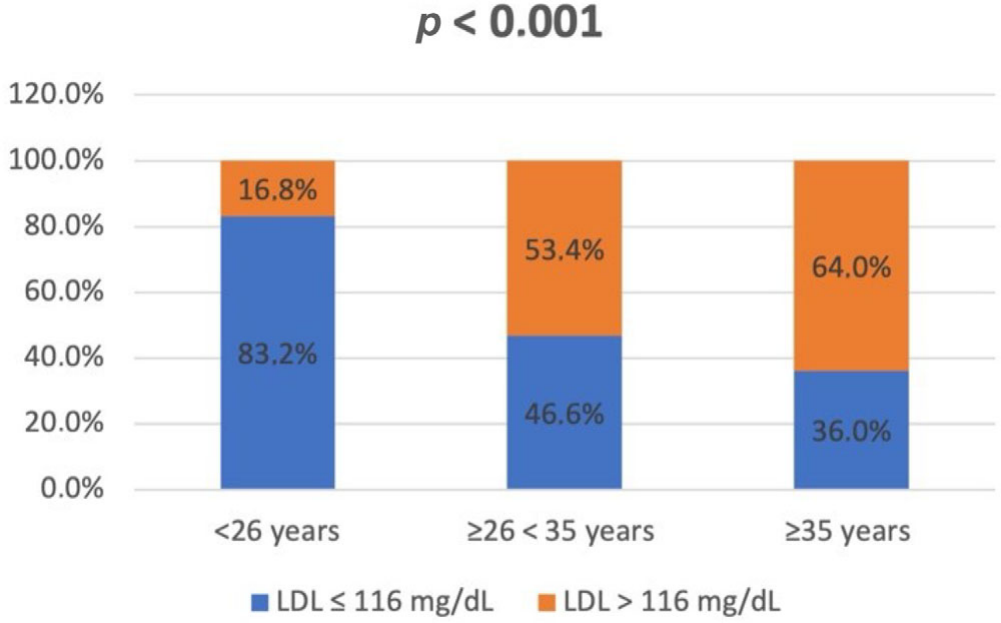
Frequency (%) of hypercholesterolemia (according to low‐density lipoprotein (LDL) concentrations, LDL > 116 mg/dL) in the three groups of patients, stratified according to the age at first visit.

After dividing the patients into three groups, based on age at first visit, different categorical variables were compared (Table 3). From group A to group B data showed an increase in absolute and relative frequencies for all the studied variables. These differences were even more evident and assumed an even greater statistical significance in the comparison between group A and group C. In particular, it emerged that from 26 years onward it was more frequent, with a particularly significant difference (*p* < 0.001), to find an increase in abdominal circumference (>94 cm), overweight and obesity (BMI ≥25 and >30 kg/cm^2^, respectively), and hypercholesterolemia (LDL > 116 mg/dL; Figures 1, 2, 3).

**TABLE 3:** Clinical characteristics of patients divided into three groups based on age at first visit. Group A < 26 years (*n* = 145, 32.6%), group B ≥ 26 ≤ 35 years (*n* = 143, 32.1%), and group C ≥ 35 years (*n* = 157, 35.3%).

	Total	Group A 1st tertile <26 years (*N* = 145)	Group B 2nd tertile ≥26 < 35 years (*N* = 143)	Group C 3rd tertile ≥35 years (*N* = 157)	*p* value
Waist circumference > 94 cm	180	27 (25.7%)	62 (54.4%)	91 (72.2%)	**<0.001**
Overweight or obesity (BMI ≥ 25 kg/m^2^)	208	34 (26.0%)	78 (60.5%)	96 (68.6%)	**<0.001**
Total testosterone deficiency (TT < 12 nmol/L)	263	70 (48.6%)	92 (64.3%)	101 (64.3%)	**0.008**
Free testosterone deficiency (cFT < 0.225 nmol/L)	196	46 (51.7%)	66 (66.7%)	82 (75.9%)	**0.002**
Altered fasting glucose (glucose 100–126 mg/dL)	24	3 (2.3%)	7 (5.3%)	14 (10.30%)	**0.024**
Insulin resistance (HOMA > 2.4)	123	23 (21.3%)	47 (39.2%)	51 (41.5%)	**0.002**
Hypercholesterolemia (LDL > 116 mg/dL)	179	21 (16.8%)	71 (53.4%)	87 (64.0%)	**<0.001**
Hypertriglyceridemia (TG > 150 mg/dL)	69	11 (7.6%)	23 (16.1%)	35 (22.3%)	**0.007**
Hypovitaminosis D (vitamin D < 75 mmol/L)	316	97 (76.4%)	103 (83.1%)	116 (89.2%)	**0.023**

Categorical variables are expressed as absolute and relative frequencies. Differences were considered statistically significant if *p* value <0.05.

Abbreviations: BMI, body mass index; cFT, calculated free testosterone; HOMA, homeostatic model assessment; LDL, low‐density lipoprotein; TT, total testosterone.

Statistically significant values with p < 0.05 are highlighted in bold.

The same comparative analysis of categorical variables among the three groups was performed for the subgroups of TRT and no‐TRT patients. While data from no‐TRT patients confirmed the results observed in the whole population (Table S3), fewer variables showed significant differences among the three age groups in the subgroup of TRT patients (Table S4). In particular, these subjects showed a high significant difference (*p* < 0.001) only in the increase in WC (>94 cm).

Among the 445 patients, almost all were azoospermic (97.98%), except for nine individuals (2.02%) in whom rare spermatozoa were detected. Of these nine patients, only one had the following parameters: semen volume of 3 mL, sperm concentration of 0.035 × 10⁶ cells/mL, and a total sperm count of 0.105 × 10⁶ cells per ejaculate. In the remaining eight patients only few sperm cells were found after centrifugation, as detailed below: 20 spermatozoa in two subjects, two spermatozoa in other three subjects, and only one spermatozoa in the remaining three subjects.

Of the total cohort, 199 patients (44.7%) underwent testicular biopsy. Among the performed TESE, 72 resulted in successful sperm retrieval and cryopreservation, with a recovery rate of 36.2%. In 34 patients (47.2%) sperm retrieval was bilateral, whereas in 38 (52.8%) it was unilateral. We further investigated the clinical characteristics of the 199 patients who underwent testicular biopsy dividing them on the basis of successful or failed sperm retrieval (Table 4). The only parameters differing between the two groups were age at first visit (*p* = 0.007) and bitesticular volume (*p* = 0.014). On this basis, we performed a binary logistic regression analysis to identify the main variable associated with sperm retrieval. Age at first visit resulted the only variable predicting successful TESE (*p* = 0.04).

**TABLE 4:** Anthropometric, hematochemical, scrotal ultrasound, and DEXA (Dual Energy X‐ray Absorptiometry) parameters of patients with non‐mosaic Klinefelter syndrome (KS) underwent testicular biopsy, divided into two groups according to the success or failure of sperm retrieval, expressed as expressed as median and 5th–95th percentiles.

	No sperm retrieval		Sperm retrieval		
	*N*	Median (5th–95th)	*N*	Median (5th–95th)	*p* value
Age at first visit	128	31.0 (18.0–44.0)	71	28.0 (16.0–41.0)	**0.007**
Waist circumference (cm)	100	93.75 (75.05–110.95)	57	93.00 (72.00–108.20)	0.382
BMI (kg/m^2^)	116	24.87 (18.59–34.12)	62	24.42 (17.46–33.06)	0.080
LH (U/L)	123	20.40 (3.98–35.48)	70	19.45 (4.93–32.37)	0.300
FSH (U/L)	123	33.20 (7.04–53.54)	70	31.85 (8.53–51.39)	0.183
TT (nmol/L)	123	10.10 (2.00–19.03)	71	10.31 (3.45–21.32)	0.113
cFT (nmol/L)	84	0.193 (0.039–0.417)	50	0.206 (0.045–0.443)	0.726
SHBG (nmol/L)	99	28.0 (13.0–59.0)	59	31.0 (15.0–60.0)	0.485
Albumin (g/L)	100	48.0 (44.1–52.0)	59	48.0 (43.0–51.0)	0.330
Estradiol (pg/mL)	119	100.0 (31.0–155.0)	66	93.0 (33.8–160.2)	0.398
Glucose (mg/dL)	117	81.0 (66.8–99.4)	67	79.0 (65.8–99.8)	0.922
Hb1Ac (%)	115	5.4 (4.8–5.9)	66	5.3 (4.8–6.0)	0.347
Insulin (mU/L)	106	9.7 (2.2–28.0)	63	7.4 (2.0–30.4)	0.542
HOMA	106	1.98 (0.51–5.39)	62	1.70 (0.56–5.04)	0.900
Total cholesterol (mg/dL)	118	179.0 (128.0–243.3)	68	177.5 (108.8–250.6)	0.511
LDL (mg/dL)	119	113.2 (74.0–181.0)	67	112.0 (59.8–180.2)	0.252
HDL (mg/dL)	117	47.0 (33.0–72.1)	66	46.5 (30.0–81.9)	0.718
Triglyceride (mg/dL)	115	86.0 (38.8–185.0)	66	72.0 (35.5–218.8)	0.554
Homocysteine (umol/L)	114	13.2 (8.1–35.2)	64	12.4 (7.5–32.2)	0.353
PTH (ng/L)	115	34.1 (14.0–88.0)	61	33.7 (13.0–88.4)	0.794
Calcium (mmol/L)	115	2.44 (2.28–2.57)	66	2.47 (2.32–2.59)	0.182
Phosphorous (mmol/L)	113	0.93 (0.66–1.34)	63	0.94 (0.71–1.33)	0.437
Vitamin D (nmol/L)	111	56.0 (24.0–100.4)	62	56.0 (22.2–101.4)	0.869
Bitesticular volume (mL)	118	3.45 (2.00–6.81)	69	4.20 (1.95–8.65)	**0.014**
Lumbar BMD	104	1.016 (0.796–1.303)	57	1.003 (0.793–1.269)	0.466
Femoral BMD	100	0.967 (0.816–1.238)	58	0.977 (0.760–1.205)	0.945

Differences were considered statistically significant if *p* value <0.05.

Abbreviations: BMD, bone mineral density; BMI, body mass index; cFT, calculated free testosterone; FSH, follicular stimulating hormone; Hb1Ac, glycosylated hemoglobin; HDL, high‐density lipoprotein; HOMA, homeostatic model assessment; LDL, low‐density lipoprotein; LH, luteinizing hormone; PSA, prostatic specific antigen; PTH, parathormone; SHBG, sex hormone binding globulin; TT, total testosterone.

Statistically significant values with p < 0.05 are highlighted in bold.

**FIGURE 4:**
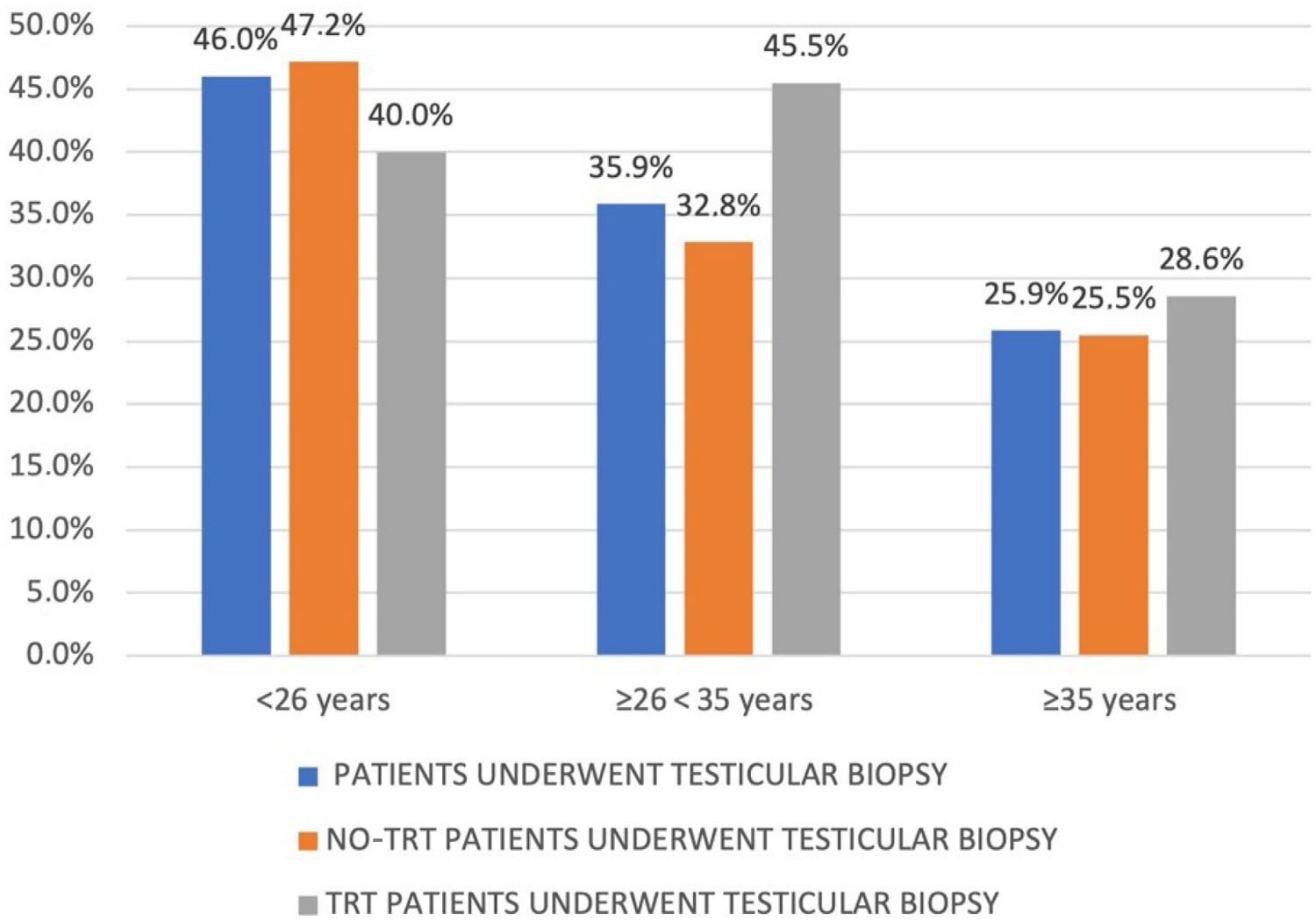
Sperm retrieval rate from testicular biopsies in three groups of patients: the whole population who underwent testicular biopsy, treated patients (testosterone replacement therapy [TRT]), and untreated patients (no TRT), stratified according to the age at first visit.

Thereafter, we further compared patients divided by age tertiles (Figure 4). In group A, 63 patients underwent testicular biopsy and 29 of them were successful, resulting in a retrieval rate of 46.0%. In group B, 78 patients underwent testicular biopsy, and 28 of them had successful retrieval (35.9%), while in group C the retrieval rate was 25.9% (15 out of 58 patients). The data indicate a 10% reduction in the retrieval rate from group A to group B and from group B to group C. However, the reduction was significant only when comparing groups A and C (*p* = 0.02). Among the 199 patients who undergone TESE, 67 subjects (33.6%) had received TRT therapy during their lifetime. No significant difference in terms of sperm retrieval rate was observed when comparing patients currently receiving testosterone therapy or who had suspended it for less than 6 months (TRT) with patients who had discontinued testosterone therapy for more than 6 months or had never received it (no TRT; *p* = 0.42). Considering age tertiles, despite a trend to reduction in the retrieval rate of the no‐TRT group, this difference did not reach a significance (*p* = 0.06).

## 4 DISCUSSION

KS is known to be characterized by testicular failure leading to infertility,^5^ alterations of glucose homeostasis,^11^ and impaired bone mineralization, resulting in reduced BMD.^12^, ^19^ Early diagnosis allows for early patient management and, if necessary, interventions such as TRT, vitamin D supplementation, and/or fertility preservation. This retrospective study, conducted on 445 patients affected by KS, took into consideration age at the first visit and the impact that it might have on the development of glycolipid and bone metabolism alterations. In the present study, the mean age at diagnosis was 24.5 ± 0.5 years, lower than the one reported by the Italian Society of Andrology and Sexual Medicine (SIAMS), which was 37.4 ± 13.4 years.^26^

The literature widely describes that patients with KS have an unfavorable metabolic phenotype, with a tendency to develop obesity, insulin resistance and type II diabetes mellitus.^18^ Among our cohort of 445 patients, 132 (29.7%) were overweight and 78 (17.5%) were obese, 24 patients (5.3%) had impaired fasting glucose, 123 (27.6%) had insulin resistance, and eight (1.7%) had diabetes. The percentage of impaired fasting glucose was higher than that reported by Bojesen et al.,^18^ while the percentage of diabetes was lower. The probability that patients with KS had overweight/obesity or a diagnosis of impaired fasting glucose, insulin resistance or diabetes increased with increasing age at first visit. The incidence of metabolic syndrome was not studied in this work, however, WC, BMI, and lipid profile were recorded. Our data showed that patients who came for the first visit later in life were more likely to be overweight or obese and had a higher probability of having increased WC, hypercholesterolemia and hypertriglyceridemia, with a significant reduction in HDL cholesterol. Indeed, patients with KS who are taken into charge later have an increased risk of having an unfavorable lipid profile. Early patient management should aim to promote an early change in lifestyle, with an increase in physical activity and the abolition of smoking habits.

The reduction of BMD and alterations of bone microarchitecture have been described in many studies regarding patients with KS.^12^, ^19^ The reduction in BMD is clinically relevant, as epidemiological studies on mortality in KS patients associated BMD reduction with an increased risk of hospitalization and death due to osteoporotic fractures.^27^ The prevalence of vertebral fractures among KS patients was studied, for the first time, by Vena et al.^21^ In their study, not a significant difference of prevalence of vertebral fractures was found between patients with normal BMD and patients with reduced BMD (15.9% vs. 13.6%; *p* = 0.800). Nevertheless, stratifying patients based on age at the time of diagnosis, the prevalence of vertebral fractures was higher in the group diagnosed >21 years of age, compared with the group diagnosed earlier. Albeit there are no screening programs for male osteoporosis yet, it is recommended to perform a DXA scan in hypogonadal patients, including those with KS, insofar as fragility fractures might occur even in patients with a normal BMD.^19^, ^28^ For this reason, the execution of DXA in KS patients is advisable, but probably not sufficient to clearly define the risk of fracture.^19^ Vertebral fractures have also been found in patients with normal testosterone values and in patients on TRT. Therefore, in addition to testosterone, other factors may play a role in the skeletal fragility and in the fracture risk, such as hypovitaminosis D, reduced INSL‐3 levels, low estrogen levels, altered AR function and sensitivity and reduced lean mass.^19^ It is known that testicular alterations, such as those present in KS, lead to vitamin D deficiency.^19^, ^29^, ^30^ For this reason, vitamin D supplementation is important in KS patients with hypovitaminosis, even in those with testosterone levels in the normal range.^19^

In our study, hypovitaminosis D was found in 316 patients (71%), with statistically significant differences between the three age groups (*p* = 0.023), 119 patients (26.7%) were defined as osteoporotic.

KS is diagnosed in 2% of the infertile male population and in 10% of azoospermic males.^31^ Until 1966, KS was considered a condition of absolute infertility; however, in that year, the first successful TESE was performed.^9^ Several studies have compared the two different TESE techniques, conventional and microsurgical. According to Sà et al.,^32^ the recovery rate using cTESE is 44% (range 16%–100%) while using mTESE is 43% (range 17%–100%). A recent meta‐analysis,^33^ conducted on 1248 patients, found no statistically significant differences comparing the two procedures. In our cohort of patients, 199 subjects (44.7%) underwent testicular biopsy using the conventional procedure. Of the 199 biopsies performed, 72 had a positive outcome, with a recovery rate of 36.2%. The retrieval rate measured in this study is similar to the one reported by Garolla et al. (34.2%)^6^ but lower than the one reported by Corona et al.,^33^ which is about 44. This difference could be due to different techniques involved; in our study all the biopsies were performed with conventional TESE, while the meta‐analysis measured the retrieval rate of biopsies performed with both conventional and microTESE.

Several studies investigated the possible testicular biopsy positive predictors factors in patients with KS. Currently, there are no markers that might indicate the positive outcome of the testicular biopsy. However, some studies have correlated sperm recovery with lower patient age,^34^ reduced period of infertility,^34^ increased testicular volume, and increased serum testosterone concentration.^35^ One recent study, conducted by Liu et al.,^36^ demonstrated that age and Anti‐Mullerian Hormon (AMH) concentrations might be the most qualified indicators of sperm recovery.

We observed that bilateral testicular volume showed a significant impact on the outcome of TESE (*p* = 0.014), suggesting a correlation between bilateral testicular volume and the success of sperm retrieval. In other words, a lower bilateral testicular volume was associated with a decreased success rate of TESE. A recent retrospective study^37^ found that positive sperm retrieval was associated with bitesticular volume >3.93 mL, left testis volume >1.79 mL, and neutrophil‐to‐lymphocyte ratio ≤1.82. This correlation, consistent with our findings, suggests that bilateral testicular volume might represent an important predictive factor for TESE outcome.

In our study, also age at first visit was a variable predicting successful TESE (*p* = 0.04). The observed recovery rates were 46% in younger patients, 35.9% in subjects aged 26–34 years, and only 25.9% in older ones. Our data show a significant difference in the retrieval rate when comparing younger and older groups (*p* = 0.02). In addition, a recent study, evaluating sperm retrieval in KS patients according to the age, reported that sperm retrieval was higher in the 20–29 years old cohort (71%) in comparison to the retrieval rate in adolescents (53%) and in the over 40 years cohort (13%).^38^ The success rate of sperm retrieval might be affected by an age‐dependent process fibrosis that occurs in the testes.^39^, ^40^

Finally, despite TRT is frequently used in KS aimed to improve andogenicity^41^ and to prevent symptoms caused by androgen deficiency, it can also impair spermatogenesis. Therefore, we also considered the effect of TRT on the sperm retrieval rate after TESE. Comparing TRT and no‐TRT patients, we observed that the retrieval rate was not significantly different (42.90% and 35.10%, respectively, *p* = 0.42).

## 5 CONCLUSIONS

Our findings support previous studies showing that early diagnosis and timely management of KS patients are crucial in preventing or reducing the associated comorbidities in later life. Indeed, we observed that glucose, lipids, and phospho‐calcium metabolism worsen with age at first visit and that the decline becomes more significant and noteworthy after the age of 26. Detecting KS at an earlier stage might potentially modify its course and improve patient outcomes. Furthermore, early management appears to be slightly associated to higher success rates in testicular biopsy for fertility potential. In fact, the retrieval rate of spermatozoa from testicular biopsies is higher in younger subjects. To enhance the early detection of KS and avoid delayed diagnosis, preventive campaign and increased awareness of the syndrome among medical professionals are of paramount importance. By promoting early identification and appropriate management, health‐care specialists might positively impact the outcomes of individuals affected by KS and mitigate the long‐term consequences associated with this condition.

## AUTHOR CONTRIBUTIONS

*Research design*: Andrea Garolla, Andrea Graziani, Giordana Ferraioli, Noemi Sagone; *Bibliographic analysis*: Andrea Graziani, Giordana Ferraioli, Noemi Sagone; *Acquisition of data*: Noemi Sagone; Analysis and interpretation of the data: Andrea Graziani, Giordana Ferraioli, Andrea Di Nisio; Writing the paper: Andrea Graziani, Giordana Ferraioli, Noemi Sagone; *Critical revision of the manuscript*: Andrea Garolla, Alberto Ferlin, Andrea Graziani, Giordana Ferraioli; *Supervision of the study*: Andrea Garolla, Alberto Ferlin. All the authors approved the submitted and final version.

## CONFLICT OF INTEREST STATEMENT

The authors declare no conflicts of interest.

## Supporting information



Supporting Information
