# Supplementary material for: Impact of age at first visit on glycolipid metabolism, bone metabolism, and fertility potential in patients with Klinefelter syndrome
Source: Andrology. 2025 Apr 7;14(1):122–31. doi: 10.1111/andr.70033 (PMC12670481; doi:10.1111/andr.70033)
Supplement: Supplementary file 1 — Supporting Information [file ANDR-14-122-s001.docx]

**Supplementary Table 1**: The table presents the characteristics of patients based on the presence of hypogonadism and the intake of replacement therapy. Columns indicate the presence or absence of hypogonadism, while rows represent treated (TRT) and untreated patients (NO TRT).

|  |  | **NO Hypogonadism** | **Hypogonadism** | **Total** |
| --- | --- | --- | --- | --- |
| **No-TRT** | **N** | 145 | 224 | 369 |
|  | **%** | 39.3% | 60.7% | 100% |
| **TRT** | **N** | 37 | 39 | 76 |
|  | **%** | 48.7% | 51.3% | 100% |
| **Total** | **N** | 182 | 263 | 445 |
|  | **%** | 41% | 59% | 100% |

*Abbreviations:* TRT: testosterone replacement therapy.

**Supplementary Table 2**: Comparison of anthropometric, haematochemical, scrotal ultrasound and DEXA parameters of KS patients on testosterone replacement therapy (TRT) and patients not on therapy (no-TRT) with non-mosaic KS, expressed as medians and 5th–95th percentiles. Differences were considered statistically significant if p value < 0.05.

|  |  | **N** | **Median (5 ^th^- 95** **^th^)** | **p value** |
| --- | --- | --- | --- | --- |
| Waist circumference (cm) | no-TRT | 293 | 96.0 (72.0-130.0) | 0.214 |
|  | TRT | 52 | 92.5 (72.7-122.2) |  |
| BMI (kg/m2) | no-TRT | 331 | 24.64 (17.78-35.81) | 0.659 |
|  | TRT | 68 | 25.25 (17.24-34.22) |  |
| LH (U/L) | no-TRT | 352 | 19.60 (8.86-37.50) | **<0.001** |
|  | TRT | 65 | 12.30 (0.10-33.56) |  |
| FSH (U/L) | no-TRT | 350 | 32.40 (14.57-59.40) | **<0.001** |
|  | TRT | 62 | 20.85 (0.20- 53.15) |  |
| TT (nmol/L) | no-TRT | 369 | 9.86 (2.15-19.44) | **<0.001** |
|  | TRT | 76 | 12.79 (2.73-31.41) |  |
| cFT (nmol/L) | no-TRT | 239 | 0.188 (0.032-0.346) | **<0.001** |
|  | TRT | 44 | 0.234 (0.044-0.841) |  |
| SHBG (nmol/L) | no-TRT | 286 | 31.0 (14.0-62.7) | **0.005** |
|  | TRT | 49 | 24.9 (9.5-64.0) |  |
| Albumin (g/L) | no-TRT | 286 | 48.0 (43.0-52.0) | 0.435 |
|  | TRT | 51 | 48.0 (44.0-54.0) |  |
| Estradiol (pg/mL) | no-TRT | 340 | 94.0 (26.1-169.9) | 0.292 |
|  | TRT | 59 | 100.0 (23.0-182.0) |  |
| Glucose (mg/dL) | no-TRT | 335 | 81.0 (65.0-101.0) | 0.495 |
|  | TRT | 60 | 83.0 (67.0-120.3) |  |
| Hb1Ac (%) | no-TRT | 326 | 5.4 (4.8-5.9) | 0.211 |
|  | TRT | 54 | 5.4 (4.8-7.2) |  |
| Insulin (mU/L) | no-TRT | 313 | 8.8 (2.0-30.0) | 0.654 |
|  | TRT | 54 | 8.8 (2.0-35.9) |  |
| HOMA | no-TRT | 312 | 1.77 (0.37-6.84) | 0.969 |
|  | TRT | 54 | 1.83 (0.42-8.13) |  |
| Total cholesterol (mg/dL) | no-TRT | 340 | 181.0 (125.1-251.0) | 0.651 |
|  | TRT | 58 | 174.0 (124.0-260.9) |  |
| LDL (mg/dL) | no-TRT | 340 | 113.0 (67.1-178.0) | 0.860 |
|  | TRT | 57 | 112.0 (69.4-184.8) |  |
| HDL (mg/dL) | no-TRT | 338 | 48.0 (32.0-71.0) | **0.010** |
|  | TRT | 57 | 42.0 (29.6-69.3) |  |
| Triglyceride (mg/dL) | no-TRT | 331 | 83.0 (40.6-206.0) | 0.603 |
|  | TRT | 58 | 94.5 (45.3-265.3) |  |
| Homocysteine (umol/L) | no-TRT | 317 | 12.6 (8.2-33.4) | 0.955 |
|  | TRT | 50 | 12.7 (7.4-38.6) |  |
| PTH (ng/L) | no-TRT | 324 | 30.4 (13.2-89.5) | 0.212 |
|  | TRT | 60 | 26.0 (10.5-96.2) |  |
| Calcium (mmol/L) | no-TRT | 335 | 2.44 (2.31-2.59) | 0.060 |
|  | TRT | 59 | 2.45 (2.27-2.59) |  |
| Phosphorous (mmol/L) | no-TRT | 319 | 0.95 (0.67-1.34) | **0.034** |
|  | TRT | 55 | 0.95 (0.60-4.04) |  |
| Vitamin D (nmol/L) | no-TRT | 316 | 56.0 (18.9-102.3) | 0.160 |
|  | TRT | 58 | 57.0 (20.0-129.2) |  |
| Bitesticular volume (mL) | no-TRT | 329 | 3.80 (2.00-7.40) | 0.129 |
|  | TRT | 59 | 3.40 (1.50-8.70) |  |
| Prostatic volume (mL) | no-TRT | 299 | 16.5 (9.0-27.0) | 0.138 |
|  | TRT | 55 | 18.5 (10.5-30.0) |  |
| Lumbar BMD | no-TRT | 292 | 0.990 (0.780-1.276) | **0.007** |
|  | TRT | 59 | 1.033 (0.780-1.341) |  |
| Femoral BMD | no-TRT | 287 | 0.967 (0.775-1.224) | **0.001** |
|  | TRT | 55 | 1.039 (0.818-1.291) |  |

*Abbreviations:* BMD: Bone Mineral Density; BMI: Body Mass Index; cFT: calculated free testosterone; FSH: follicular stimulating hormone; Hb1Ac: glycosylated haemoglobin; HOMA: homeostatic model assessment; LH: luteinizing hormone; SHBG: sex hormone binding globulin; PTH: parathormone; TT: total testosterone.

**Supplementary Table 3**: Clinical characteristics of untreated patients divided into 3 groups based on age at first visit. Categorical variables of these patients are expressed as absolute and relative frequencies. Differences were considered statistically significant if p value < 0,05.

|  | **Total** | Group A  1st tertile  <26 years | Group B  2nd tertile  ≥26, <35 years | Group C  3rd tertile  ≥35 years | **p value** |
| --- | --- | --- | --- | --- | --- |
| **Waist circumference >94 cm** | 156 | 25 (28.4%) | 51 (54.3%) | 80 (72.1%) | **< 0.001** |
| **Overweight or obesity**  **(BMI ≥25 kg/m2)** | 171 | 27 (25.2%) | 62 (59.6%) | 82 (67.8%) | **< 0.001** |
| **Total Testosterone deficiency (TT< 12 nmol/L)** | 224 | 59 (50.0%) | 80 (68.0%) | 85 (63.9%) | **0.013** |
| **Free Testosterone deficiency (cFT< 0,225 nmol/L)** | 168 | 37 (51.4%) | 59 (69.4%) | 72 (76.6%) | **0.002** |
| **Altered fasting glucose**  **(glucose 100-126 mg/dL)** | 19 | 2 (1.9%) | 6 (5.4%) | 11 (9.30%) | 0.055 |
| **Insulin-resistance**  **(HOMA > 2.4)** | 103 | 19 (21.6%) | 40 (38.5%) | 44 (40.4%) | **0.012** |
| **Hypercholesterolemia**  **(LDL > 116 mg/dL)** | 154 | 17 (16.3%) | 62 (54.9%) | 75 (62.5%) | **< 0.001** |
| **Hypertriglyceridemia**  **(TG > 150 mg/dL)** | 56 | 9 (7.6%) | 20 (16.9%) | 27 (20.3%) | 0.059 |
| **Hypovitaminosis D**  **(vitamin D < 75 mmol/L)** | 264 | 80 (75.5%) | 82 (79.6%) | 102 (90.3%) | **0.013** |

*Abbreviations:* BMI: Body Mass Index; cFT: calculated free testosterone; HOMA: homeostatic model assessment; TT: total testosterone.

**Supplementary Table 4**: Clinical characteristics of treated patients divided into 3 groups based on age at first visit. Categorical variables of these patients are expressed as absolute and relative frequencies. Differences were considered statistically significant if p value <0,05.

|  | **Total** | Group A  1st tertile  <26 years | Group B  2nd tertile  ≥26, <35 years | Group C  3rd tertile  ≥35 years | **P value** |
| --- | --- | --- | --- | --- | --- |
| **Waist circumference >94 cm** | 24 | 2 (11.8%) | 11 (55.0%) | 11 (73.3%) | **< 0.001** |
| **Overweight or obesity**  **(BMI ≥25 kg/m2)** | 37 | 7 (29.2%) | 16 (64.0%) | 14 (73.7%) | **0.007** |
| **Total Testosterone deficiency (TT< 12 nmol/L)** | 38 | 11 (42.3%) | 11 (44.0%) | 16 (66.7%) | 0.163 |
| **Free Testosterone deficiency (cFT< 0,225 nmol/L)** | 26 | 9 (52.9%) | 7 (50.0%) | 10 (71.4%) | 0.454 |
| **Altered fasting glucose**  **(glucose 100-126 mg/dL)** | 5 | 1 (4.5%) | 1 (5.0%) | 3 (16.7%) | 0.310 |
| **Insulin-resistance**  **(HOMA > 2.4)** | 18 | 4 (20.0%) | 7 (43.8%) | 7 (50.0%) | 0.147 |
| **Hypercholesterolemia**  **(LDL > 116 mg/dL)** | 25 | 4 (19.0%) | 9 (45.0%) | 12 (75.0%) | **0.003** |
| **Hypertriglyceridemia**  **(TG > 150 mg/dL)** | 13 | 2 (7.7%) | 3 (12.0%) | 8 (33.3%) | 0.060 |
| **Hypovitaminosis D**  **(vitamin D < 75 mmol/L)** | 52 | 17 (81.0%) | 21 (100.0%) | 14 (82.4%) | 0.110 |

*Abbreviations:* BMI: Body Mass Index; cFT: calculated free testosterone; HOMA: homeostatic model assessment; TT: total testosterone.
